# Pre-Referral Antibiotic Use and Bacteriology in Children with Persistent Otitis Media with Effusion Undergoing Ventilation Tube Insertion in Korea

**DOI:** 10.3390/jcm15218635

**Published:** 2026-11-05

**Authors:** Inho Yoon, Junsung Suh, Jin Kim, Min Young Kwak, Hye Ah Joo

**Affiliations:** 1 Sanbon Clear Neurology Clinic, Gunpo 15865, Republic of Korea; inoinoy03@gmail.com; 2 Department of Otorhinolaryngology-Head and Neck Surgery, Dongtan Sacred Heart Hospital, Hallym University College of Medicine, Hwaseong 18450, Republic of Korea; csjohn93@hallym.or.kr (J.S.); jinsound@hallym.or.kr (J.K.); drminmin@hallym.or.kr (M.Y.K.)

**Keywords:** otitis media with effusion, middle ear effusion, ventilation tube, antibiotics, Republic of Korea

## Abstract

**Background**: Current clinical guidelines for pediatric otitis media with effusion (OME) advise watchful waiting and strongly discourage systemic antibiotic use. This study evaluated the rate of pre-referral antibiotic use among children with persistent OME requiring ventilation tube (VT) insertion and analyzed the bacterial culture positivity of middle ear effusion. **Methods**: We retrospectively reviewed 38 children (56 ears) aged 3 months to 12 years with persistent OME (≥3 months) who underwent first-time VT insertion between March 2025 and August 2026 at a secondary referral center. Prior antibiotic history from primary care clinics was investigated, and antibiotics were discontinued upon referral. Middle ear effusion samples were aseptically aspirated during VT insertion for bacterial direct culture and susceptibility testing. **Results**: Among the 38 children, 78.9% had received antibiotics prescribed by primary care clinics for OME before referral. Following a mean antibiotic-free interval of 27.34 days, direct bacterial cultures of middle ear effusion yielded positive growth in only 10 of 56 ears (17.9%). The most commonly isolated organisms were coagulase-negative staphylococci (CoNS; 80%, 8/10), of which 75% (6/8) showed methicillin resistance. Prior antibiotic exposure was not significantly associated with bacterial culture positivity (18.2% vs. 16.7%, Fisher’s exact test, *p* = 1.000). **Conclusions**: Despite guideline recommendations, pre-referral antibiotic use remains common among children with OME requiring VT insertion in South Korea. Given the observational design, small sample size, and culture sensitivity limits, these findings support adherence to existing clinical guidelines. Educational initiatives and guidance are needed to promote evidence-based prescribing in primary care.

## 1. Introduction

Otitis media with effusion (OME) is a condition characterized by the accumulation of fluid in the middle ear in the absence of signs and symptoms of acute inflammation, such as fever or otalgia [1]. The primary etiology of OME in children is attributed to the anatomical and functional immaturity of the Eustachian tube, which lies more horizontally in children than in adults, along with the underdevelopment of the muscular structure responsible for its active opening [2]. Additional contributing factors, such as biofilm formation due to viral infections, feeding practices, pacifier use, and adenoid vegetation, may cause Eustachian tube dysfunction and consequently lead to OME. Acute otitis media (AOM), a closely related condition, is an acute infectious (bacterial and/or viral) process, and OME is often considered a sequela of AOM, resulting from inflammatory mediators and cytokines following infection [3].

The clinical course of OME is typically prolonged, often lasting for several months and recurring throughout childhood. OME has significant medical, social, and economic consequences, including hearing loss, changes in the tympanic membrane, and an adverse effect on the quality of life of affected children and their families [4].

According to the Korean clinical practice guidelines on otitis media in children, it is recommended that clinicians manage children with OME according to an observation policy for at least 3 months from onset, unless high-risk factors are present [5]. These high-risk factors include: (1) sensorineural hearing loss independent of OME; (2) uncorrectable visual impairment; (3) Down syndrome or craniofacial anomalies; (4) cleft palate; (5) autism spectrum disorder or pervasive developmental disorder; (6) suspected or diagnosed speech and language delay; and (7) developmental or other cognitive impairments. The use of medications, including antibiotics, oral and nasal steroids, antihistamines, and decongestants, is discouraged. The decision for surgical intervention is made after 3 months of observation, following an evaluation of hearing, unless there are irreversible changes in the tympanic membrane. Ventilation tube (VT) insertion is the preferred initial surgical procedure, and adenoidectomy may be performed concurrently with VT insertion in recurrent cases requiring repeated surgical intervention. The clinical practice guideline developed by the American Academy of Otolaryngology—Head and Neck Surgery Foundation shares the same view and strongly recommends against the use of systemic antibiotics for treating OME [6]. The Cochrane review and international consensus also reported that the evidence supporting the use of antibiotics for OME is of low to very low certainty [7,8]. The Korean guidelines do not provide recommendations regarding the use of complementary and alternative medicine for the treatment of OME [5].

However, based on clinical experience at the otology clinic of a secondary referral center in South Korea, the authors observed that many children with persistent OME are frequently prescribed inappropriate, prolonged, and broad-spectrum antibiotics by primary care providers in pediatrics and otolaryngology prior to appropriate surgical intervention. This observation raised concerns regarding current antibiotic prescription practices at primary care clinics for pediatric OME in South Korea, and it was considered necessary to investigate whether these prescriptions contribute to the resolution of persistent middle ear effusion through bacteriological analysis. Therefore, the present study aimed to evaluate the rate of antibiotic use prescribed at primary care clinics among children with prolonged OME requiring VT insertion, as well as bacterial culture positivity in middle ear fluid obtained aseptically during VT insertion.

## 2. Materials and Methods

This retrospective study was conducted in the otolaryngology department of a secondary referral center in South Korea. A total of 38 children (56 ears) diagnosed with OME who underwent VT insertion by a single surgeon (H.A.J.) were reviewed from March 2025 to August 2026. The study included patients aged 3 months to 12 years with persistent OME, defined as middle ear effusion persisting for 3 months or longer, who met the criteria for myringotomy with first-time VT insertion. All selected cases were diagnosed with OME based on otoscopic examination and impedance audiometry, which demonstrated a type B tympanogram. At their first outpatient visit, parents were asked about the child’s previous symptoms, diagnosis, follow-up period, and any antibiotic treatment prescribed at primary care clinics specifically for persistent OME, excluding cases in which antibiotics had been prescribed for concomitant or recent AOM, upper respiratory infections, or other infections. Based on the final diagnosis of OME, patients were instructed to discontinue oral antibiotics reportedly prescribed for OME until surgery, as part of standard clinical care and independently of this study. A retrospective chart review was conducted to analyze the rate of antibiotic prescriptions at primary care clinics in children with prolonged OME requiring VT insertion. The period between the initial diagnosis and VT insertion during which antibiotics were discontinued was also reviewed retrospectively.

**Figure 1:**
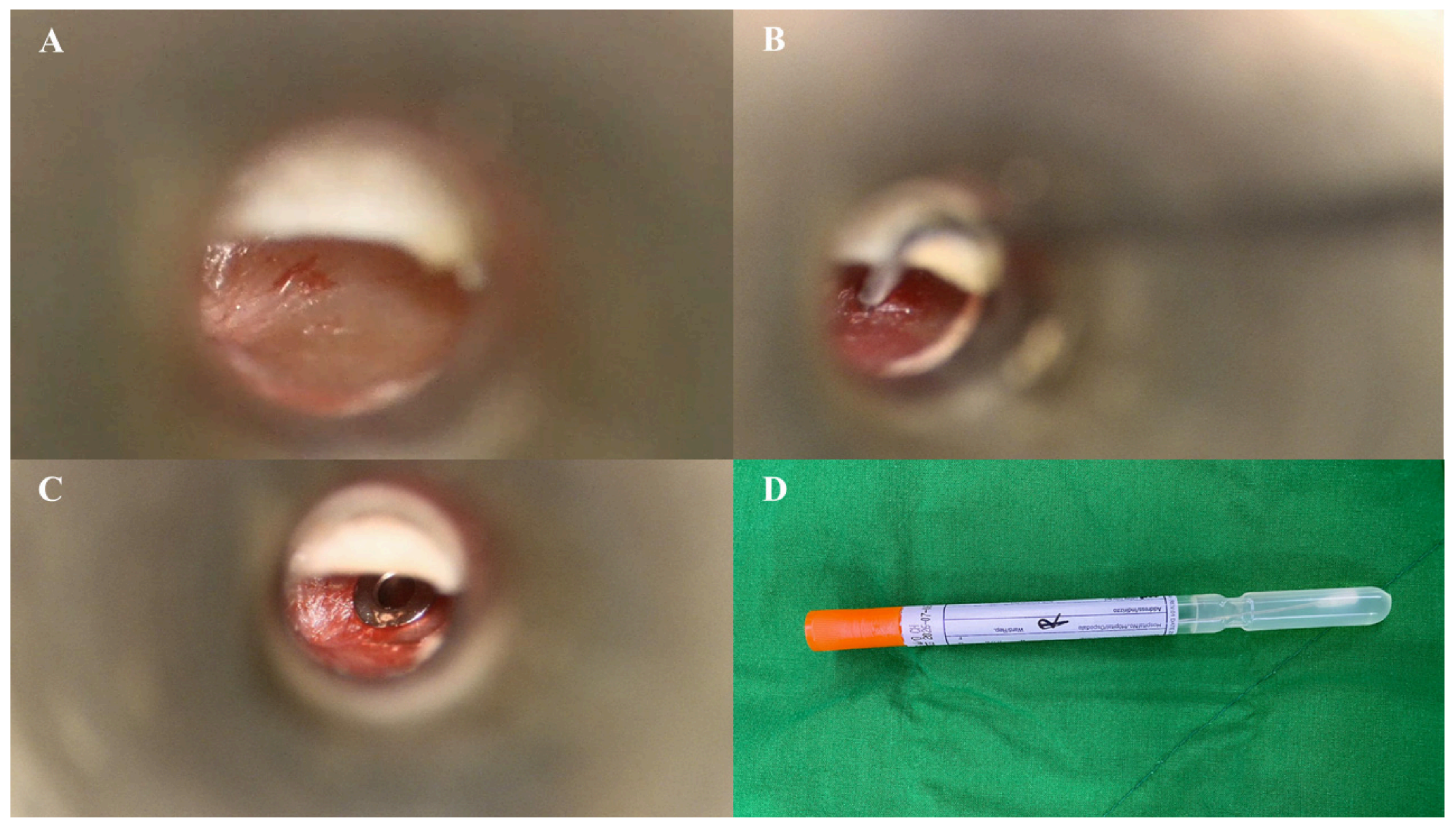
The process of ventilation tube insertion for right otitis media with effusion. (**A**) Under general anesthesia, the auditory canal was cleansed with normal saline and potadine, and cerumen was removed. (**B**) After a myringotomy, the effusion was aspirated using a 1cc syringe fitted with a 25-gauge fine long needle. (**C**) Following suction and irrigation of the residual middle ear effusion, a ventilation tube was placed in the anteroinferior quadrant of the tympanic membrane. (**D**) The effusion sample collected for direct culture was placed in Stuart transport medium.

During the VT insertion procedure, middle ear effusion samples were routinely collected from each OME patient before inserting the tube and sent for direct culture. The process is shown in detail in Figure 1. The surgery was performed under general anesthesia with mask ventilation administered by an anesthesiologist, using microscopy. The patient’s external auditory canal was cleansed with normal saline and potadine irrigation, and cerumen was carefully removed, taking care to avoid bleeding. After a myringotomy was made in the anteroinferior portion of the tympanic membrane, the effusion was aseptically aspirated using a 1cc syringe fitted with a 25-gauge fine long needle. Following suction of the residual middle ear effusion and adequate irrigation, VT was inserted and bleeding was controlled with a Bosmin gauze ball. Each sample collected for direct culture was placed in Stuart transport medium and sent to the microbiology laboratory for analysis. The samples were then inoculated onto blood agar and into thioglycollate liquid medium. All cultures were incubated at 35 °C for at least 24 h, and the resultant bacteria were identified by Gram staining and biochemical tests. Antibiotic susceptibility testing was performed after bacterial identification. The results of direct cultures of middle ear effusion samples were retrospectively analyzed. The accuracy of the tests conducted in the hospital laboratory was guaranteed.

This study protocol was approved by the Institutional Review Boards (IRBs) (No. 2025-07-018) and was conducted in accordance with the principles of the Declaration of Helsinki. The initial IRB approval was obtained on 28 August 2025 for the retrospective review of clinical records of patients treated between March 2025 and July 2025. Subsequently, continuing review and protocol amendment approval were obtained on 21 August 2026, to extend the study period through August 2026, covering the retrospective review of clinical records of patients treated between March 2025 and August 2026. The IRB waived the requirement for informed consent because of the minimal risk associated with the retrospective review of clinical data.

Continuous variables were presented as mean ± standard deviation, and categorical variables as frequency and percentage. Fisher’s exact test was used to assess the association between previous antibiotic use and bacterial culture positivity in middle ear effusion. Odds ratios (ORs) with exact 95% confidence intervals (CIs) were also calculated. Statistical analyses were performed using SPSS software version 24.0 (IBM Corp., Armonk, NY, USA). A *p*-value less than 0.05 was considered statistically significant.

## 3. Results

Clinical characteristics of patients with OME and ears undergoing VT insertion are presented in Table 1. Among 38 children with OME (24 boys and 14 girls), 56 ears underwent VT insertion, including bilateral insertion in 18 children and unilateral insertion in 20. The mean age at the initial outpatient visit was 50.63 months, and approximately 79% of the children had a history of antibiotic use prescribed by primary care clinics for prolonged OME. Oral antibiotics reportedly prescribed for persistent OME were discontinued at the initial visit, and the mean interval from the initial visit to VT insertion (antibiotic withdrawal period) was 27.34 days. At the time of VT insertion, all included ears had normal-appearing tympanic membranes without redness or perforation, with middle ear effusion as the only abnormal finding.

Middle ear effusion samples were collected from all 56 ears. Serous and mucoid effusions accounted for 33.9% and 66.1% of the samples, respectively. Direct culture of middle ear effusion yielded positive results in 10 samples (17.9%), and the bacterial isolates are presented in Table 2. The most commonly identified organisms were coagulase-negative staphylococci (CoNS) (8 isolates, 80%), followed by one methicillin-sensitive *Staphylococcus aureus* (MSSA) isolate and one *Streptococcus mitis* isolate. Among the CoNS isolates, methicillin resistance was detected in 6 isolates.

At the ear level, there was no significant association between previous antibiotic use and bacterial culture positivity in middle ear effusion (Fisher’s exact test, *p* = 1.000; OR, 1.11; 95% CI, 0.20–6.09). Culture positivity was observed in 8 of 44 ears (18.2%) with a history of antibiotic use and in 2 of 12 ears (16.7%) without previous antibiotic use. To account for the potential lack of independence between ears from the same patient, an additional patient-level analysis was performed, including all 38 patients. Similarly, at the patient level, there was no significant association between previous antibiotic use and bacterial culture positivity in middle ear effusion (Fisher’s exact test, *p* = 1.000; OR, 0.91; 95% CI, 0.15–5.58). Culture positivity was observed in 7 of 30 patients (23.3%) with a history of antibiotic use and in 2 of 8 patients (25.0%) without previous antibiotic use.

## 4. Discussion

This study evaluated antibiotic use among children with prolonged OME requiring VT insertion, and the prevalence of positive bacterial cultures in middle ear fluid using data from a secondary referral center. Among the children referred for VT insertion, 78.9% had previously received antibiotics despite the lack of an indication according to current clinical guidelines. Following a mean antibiotic-free interval of 27.34 days after the initial diagnosis, bacterial cultures were positive in only 17.9% of middle ear effusion samples. These findings suggest a potential discrepancy between guideline recommendations and reported pre-referral antibiotic use among children with persistent OME referred to our center for VT insertion in South Korea.

Conventional bacterial culture and highly sensitive molecular detection techniques have shown that the healthy middle ear is typically a sterile site [9]. In the present study, the bacterial culture positivity rate was relatively low (17.9%). Previous studies have reported positive bacterial cultures in approximately one-third of patients with OME, with *Streptococcus pneumoniae*, *Haemophilus influenzae*, and *Moraxella catarrhalis* being the most frequently identified species [1]. Although bacterial involvement has been implicated in the pathogenesis of OME, several studies have shown that antibiotic therapy does not confer long-term benefits in terms of hearing outcomes, the need for VT insertion, or speech and language development [7]. Furthermore, unnecessary antibiotic use contributes to the emergence of antimicrobial-resistant pathogens.

Although most children in our cohort had previously received antibiotics, our study was not designed to determine whether prior antibiotic exposure contributed to the emergence or selection of antimicrobial-resistant organisms. In addition, no statistically significant association was observed between previous antibiotic use and bacterial culture positivity. This finding should be interpreted cautiously, as the relatively small sample size and limited number of culture-positive samples may have reduced the statistical power to detect an association. Therefore, the lack of a statistically significant association should not be interpreted as evidence that previous antibiotic use has no relationship with bacterial culture positivity. Nevertheless, these findings, together with previous evidence showing limited long-term benefits of antibiotic therapy for OME, suggest that the clinical benefits of antibiotic use in this setting may be limited.

In the present study, the most commonly isolated organisms were CoNS, and 6 of the 8 isolates exhibited methicillin resistance. Although CoNS are commonly regarded as non-pathogenic commensals, they are well known for their ability to form biofilms, making them a leading cause of biomaterial-related infections [10]. A previous study reported that CoNS accounted for 60% of bacterial isolates in OME, suggesting that these organisms may be relevant to the microbiology of OME [11]. In our study, precautions were taken to minimize contamination from the normal flora of the external auditory canal during specimen collection. Prior to myringotomy, the external auditory canal was rigorously cleansed with saline and povidone-iodine irrigation under microscopic guidance, and middle ear effusion was directly aspirated through an intact tympanic membrane using a fine 25-gauge needle without contacting the canal wall. These precautions support the possibility that some of the isolated CoNS may represent true middle ear colonizers rather than contaminants. Nevertheless, contamination cannot be completely excluded, and the detection of CoNS, including methicillin-resistant isolates, does not necessarily indicate active infection or clinically significant colonization. Because molecular microbiological investigations were not performed, we could not definitively distinguish true middle ear colonization or infection from contamination. Therefore, the role of CoNS in persistent OME in our cohort remains uncertain and should be interpreted cautiously.

South Korea has a universal National Health Insurance system that provides broad access to healthcare at relatively low out-of-pocket costs. In addition, the healthcare system allows patients to directly access a wide range of community-based clinics and hospitals without a strict primary care gatekeeping system. Patients can even visit multiple healthcare facilities on the same day if needed. Consequently, children can readily receive medical consultations, diagnoses, and prescriptions from numerous healthcare facilities in their neighborhoods.

Notably, 78.9% of children with OME in our study had previously received antibiotics despite the lack of a guideline-based indication. This finding is consistent with previous reports of inappropriately high antibiotic use among children in South Korea, including excessive use of broad-spectrum antibiotics [12]. This pattern of overuse and misuse among pediatric outpatients extends beyond acute respiratory tract infections to the routine management of otitis media. In a population-based study using the national insurance reimbursement database, the proportion of pediatric patients prescribed antibiotics was 1.5–5.7 times higher in South Korea than in European countries, and 78.5% of the antibiotics prescribed were broad-spectrum agents, compared with 50% in the USA and 63% in Germany [13]. Of note, regarding changes in the bacterial isolation rate of otitis media in South Korea, the detection rate of methicillin-resistant *Staphylococcus aureus* (MRSA) has steadily increased, a phenomenon likely exacerbated by frequent, prolonged antibiotic exposure [1,14]. These concerns are particularly relevant given that relatively few policies or antimicrobial stewardship programs have been implemented to promote appropriate antibiotic use in pediatric outpatient settings. Strengthening such efforts is important not only to improve antibiotic prescribing practices but also to address the broader clinical challenge of antimicrobial resistance across different patient populations and healthcare settings [15].

This study has several limitations. First, because this study was conducted at a single secondary referral center with a relatively small cohort, our findings may not fully represent nationwide trends, limiting the generalizability. The small sample size and the limited number of culture-positive samples also reduced the statistical power to detect potential associations between previous antibiotic use and bacterial culture positivity. Moreover, all participants had persistent OME requiring first-time VT insertion, representing a selected population with more prolonged disease than the broader population of children with OME, including those whose effusion resolves spontaneously. Second, antibiotic prescription history and medication use were assessed based on parental reports obtained during outpatient visits, which may have introduced recall bias. Detailed information on antibiotic classes, treatment duration, and the number of treatment courses or repeated prescriptions was not systematically collected and analyzed. In South Korea, although a nationwide drug utilization review (DUR) system is available to identify duplicate prescriptions, drug–drug interactions, and contraindicated medications in outpatient settings [16], it is difficult for individual physicians to routinely track a patient’s complete history of antibiotic prescriptions. Therefore, implementing a nationwide system that enables physicians to access patients’ previous prescription records or medication history would facilitate more accurate research and improve the reliability of future studies. Detailed information on the type and duration of prescribed antibiotics should also be incorporated into future studies. Third, the bacterial culture yield from middle ear fluid obtained during VT insertion was relatively low in this study. It remains unclear whether this low detection rate was attributable to previous antibiotic use or reflected the microbiological status of the middle ear. Although there was an antibiotic-free interval between the initial outpatient visit and surgery, the potential effects of previous antibiotic exposure on bacterial recovery cannot be excluded. Furthermore, conventional culture methods may not detect all bacteria, particularly biofilm-associated or non-culturable organisms. None of the patients developed acute inflammatory signs after antibiotic discontinuation, and middle ear effusion persisted with a culture positivity rate of 17.9%. However, these observations do not establish that persistent OME was maintained primarily by Eustachian tube dysfunction rather than bacterial factors. The low culture positivity rate should therefore be interpreted cautiously and does not exclude a potential bacterial contribution to persistent OME. Our findings do not directly establish the effectiveness or ineffectiveness of antibiotic therapy but highlight the need for further studies to clarify the role of bacteria in persistent OME and the clinical implications of antibiotic use. Finally, microbiological assessment was restricted to conventional direct culture of middle ear fluid. Therefore, we could not definitively distinguish true middle ear infection or colonization from contamination, particularly for CoNS, which may originate from the normal flora of the skin or external auditory canal. Incorporating molecular diagnostic modalities, such as polymerase chain reaction (PCR) and other complementary microbiological approaches, may provide greater sensitivity and additional insights into non-culturable or biofilm-associated pathogens associated with persistent OME.

Future studies should include large, multi-center investigations using representative populations and systematic prescription data to further evaluate the current status of pediatric OME management in South Korea. Moreover, proactive dissemination and clinical adherence to evidence-based OME clinical practice guidelines among primary care physicians may reduce inappropriate antibiotic prescribing in children and ensure optimal management.

## 5. Conclusions

In conclusion, our findings demonstrated a high rate of antibiotic use in children with OME requiring VT insertion, which is inconsistent with current clinical guidelines. Despite frequent antibiotic use prior to referral, bacterial cultures were positive in only 17.9% of ears following discontinuation of antibiotic use, with CoNS being the most commonly isolated organisms. Previous antibiotic use was not significantly associated with bacterial culture positivity in middle ear effusion. These findings highlight the need for wider dissemination and education on evidence-based OME management guidelines among primary care physicians in Korea. However, given the observational design, small sample size, and limitations of conventional culture methods, our results support adherence to existing guidelines but do not independently establish the effectiveness of antibiotic discontinuation or clarify the underlying mechanisms of antimicrobial resistance. Further large-scale, multicenter studies are warranted to validate these findings and better characterize the microbiological features of pediatric OME.

## Figures and Tables

**Table 1:** Clinical characteristics of patients with otitis media with effusion and ears undergoing ventilation tube insertion.

Variables	
No. of patients	38
Age at initial visit (months)	50.63 ± 33.30
Sex	
Male	24 (63.2)
Female	14 (36.8)
History of antibiotic use	
Yes	30 (78.9)
No	8 (21.1)
Time from initial visit to VT insertion (days)	27.34 ± 19.18
No. of ears	56
Side	
Right	32 (57.1)
Left	24 (42.9)
Positive culture results	10 (17.9)
Middle ear fluid characteristics	
Serous	19 (33.9)
Mucoid (glue-like)	37 (66.1)

The data are presented as mean ± standard deviation, frequency and percentage, unless otherwise noted. VT, ventilation tube.

**Table 2:** Isolated bacterial species of middle ear fluid from patients with otitis media with effusion.

Organism		No. (%) of Isolates
Total		56 (100)
No growth		46 (82.1)
Growth		10 (17.9)
Gram (+)		
	Coagulase-negative staphylococci (CoNS)	8 (80.0)
	Methicillin-resistant CoNS	6 (60.0)
	Methicillin-sensitive CoNS	2 (20.0)
	Methicillin-sensitive *Staphylococcus aureus* (MSSA)	1 (10.0)
	*Streptococcus mitis*	1 (10.0)

## Data Availability

The datasets generated during and/or analyzed during the current study are available from the corresponding author on reasonable request.

## Abbreviations

The following abbreviations are used in this manuscript:

AOM	Acute otitis media
CI	Confidence interval
CoNS	Coagulase-negative staphylococci
IRB	Institutional Review Board
MRSA	Methicillin-resistant *Staphylococcus aureus*
MSSA	Methicillin-sensitive *Staphylococcus aureus*
OME	Otitis media with effusion
OR	Odds ratio
VT	Ventilation tube
